# Palonosetron versus tropisetron with dexamethasone for prevention of postoperative nausea and vomiting in pediatric adenotonsillectomy: a single center, randomized controlled trial

**DOI:** 10.3389/fmed.2025.1549619

**Published:** 2025-08-01

**Authors:** Hualin Chen, Liping Sun, Jijian Zheng, Hongbin Gu

**Affiliations:** Department of Anesthesiology, Shanghai Children’s Medical Center, Shanghai Jiao Tong University School of Medicine, Shanghai, China

**Keywords:** PONV, palonosetron, tropisetron, children, adenotonsillectomy

## Abstract

**Background:**

Postoperative nausea and vomiting (PONV) remain significant complications following pediatric adenotonsillectomy. Although palonosetron, a second-generation 5-HT_3_ receptor antagonist, has shown potential for preventing PONV, further research is needed to assess its efficacy when combined with dexamethasone in pediatric otolaryngologic procedures. This study aims to observe the efficacy of palonosetron or tropisetron combined with dexamethasone in preventing PONV in children undergoing adenotonsillectomy.

**Methods:**

We enrolled 110 children scheduled for elective adenotonsillectomy under general anesthesia. Based on a computer-generated random number table, children were assigned in equal proportions to receive either palonosetron or tropisetron. The P-group received palonosetron (1 μg/kg, maximum 50 μg), while the T-group received tropisetron (0.1 mg/kg, maximum 2 mg) 5 min before anesthesia induction. After receiving their allocated drug, children received intravenous dexamethasone (0.15 mg/kg, maximum 5 mg) during induction. Primary outcomes included PONV incidence at 6, 24, and 48 h postoperatively. Secondary outcomes included the number of children with PONV scores of 1, 2, and 3, rescue antiemetic medication, and adverse events.

**Results:**

A total of 110 children (aged 3–12 y, ASA physical status 1 or 2, weighted 14.3–47.3 kg) were enrolled in the study between December 2021 and July 2023. The P-group (*n* = 52) demonstrated significantly lower PONV incidence than the T-group (*n* = 51) during the 0–48 h (5.8% vs. 25.5%, *p* < 0.05). Notably, this difference was most pronounced during the first 24 h: 0–6 h (0% vs. 10%, *p* < 0.05) and 6–24 h (3.8% vs. 7.8%, *p* < 0.05). The difference during 24–48 h was insignificant (4% vs. 8%, *p* > 0.05). Transient junctional rhythm occurred in three patients receiving tropisetron, which did not affect circulation. Headache and dizziness were similar between groups within 48 h (P-group: 3.8%, T-group: 7.8%, *p* > 0.05).

**Conclusion:**

In pediatric adenotonsillectomy, palonosetron with dexamethasone gives better PONV prevention than tropisetron with dexamethasone, especially in the first 24 h, with comparable safety.

**Clinical trial registration:**

https://www.chictr.org.cn/showproj.html?proj=127115, ChiCTR2100046848.

## Introduction

PONV remains one of the most challenging complications in pediatric adenotonsillectomy, with reported incidence rates as high as 89% in the absence of prophylaxis (1). Beyond causing significant patient distress, PONV can lead to multiple adverse consequences, including prolonged hospital stays, increased healthcare costs, and a substantial burden on both healthcare systems and families.

While various preventive strategies exist (total intravenous anesthesia, non-opioid multi-modal pain management, shorter preoperative fasting time, carbohydrate loading, Hydration, Transcutaneous electrical acupoint atimulation) (2), pharmacological prophylaxis remains the cornerstone of PONV management. 5-hydroxytryptamine type 3 (5-HT_3_) receptor antagonists have emerged as a primary prophylactic option, operating through a dual mechanism: inhibiting 5-HT release from gastrointestinal chromaffin cells and blocking signal transmission to the chemoreceptor trigger zone. Current clinical guidelines recommend combining 5-HT_3_ receptor antagonists with low-dose dexamethasone for high-risk pediatric patients (3).

Despite this combination approach, first-generation 5-HT_3_ antagonists such as tropisetron have shown limited success, with PONV rates remaining around 28.9% in pediatric adenotonsillectomy (4). Palonosetron, a second-generation 5-HT_3_ receptor antagonist, offers potential advantages through its distinctive pharmacological profile: highly selective, competitive, high-affinity antagonist of the 5-HT_3_ receptor and an extended half-life of approximately 40 h (5). Palonosetron (0.5–1.5 μg/kg) has demonstrated promising results in pediatric strabismus surgery, with PONV rates of only 20% over 48 postoperative hours (6). However, evidence for its efficacy in pediatric adenotonsillectomy remains limited.

We conducted this randomized, double-blind trial to evaluate whether palonosetron plus dexamethasone provides superior PONV prophylaxis compared to tropisetron plus dexamethasone in children undergoing adenotonsillectomy. We hypothesized that palonosetron’s unique pharmacological properties would improve clinical outcomes in this high-risk PONV population.

## Materials and methods

This double-blinded, randomized controlled trial has been approved by the ethics committee of our hospital (SCMCIRB-K2021004-1) and registered at the China Clinical Trials Registry in May 2021 with registration number ChiCTR2100046848. Written informed consent was obtained from all participants’ parents or legal guardians. This study adhered to the principles of the Declaration of Helsinki and Good Clinical Practice (GCP) guidelines. Inclusion criteria: children in our center (aged 3 to 12 years with an American Society of Anesthesiology physical status of 1 or 2) scheduled for elective adenotonsillectomy under general anesthesia with tracheal intubation were included in the study. Exclusion criteria comprised: history of severe PONV or motion sickness, antiemetic use within 24 h before surgery, known allergies to study medications, American Society of Anesthesiology physical status >2, significant cardiovascular, hepatic, or renal disease, body mass index >30 kg/m^2^, diabetes mellitus, sickle cell disease, known coagulation disorders, hypertension and prolonged Q-Tc interval on electrocardiogram, inability of the child or parents to communicate effectively, peptic ulcer, bleeding disorders.

Patients were allocated into either P-group or T-group using a computer-generated random number table (SPSS Inc., USA) before commencing the study, with each group including 55 patients at a 1:1 ratio. A separate investigator maintained sequentially numbered, opaque, sealed envelopes that concealed group assignments. An independent researcher prepared all the study drugs. The envelopes were opened before the induction of anesthesia by a designated researcher. The researcher then prepared the appropriate study medication as an injectable solution (5 mL) placed in identical syringes for the induction of anesthesia. To ensure blinding, the study drugs were of the same color and syringe size and were dispensed in similar containers, making them indistinguishable. All patients and investigators collecting the postoperative data were blinded to the group allocation in adherence to the principles of blinding. The blind principle would be set aside by the critical need for immediate treatment and accurate reporting when a serious adverse event such as anaphylaxis occurs.

Routine preoperative examinations (blood routine, coagulation function, liver and kidney function, chest X-ray, and electrocardiogram) were conducted on all enrolled children. Patients were instructed to fast for 6 h and abstain from water for 2 h before surgery. Upon arrival in the operating room, standard monitoring was applied to all patients, including heart rate, blood pressure, pulse oximetry, and electrocardiogram. An intravenous infusion of Ringer’s acetate solution was administered using the “4:2:1” formula. The experimental drugs were administered by the primary anesthesiologist 5 min before anesthesia induction. The P-group received palonosetron [(1 μg/kg, maximum 50 μg), (Qilu Pharmaceutical (Hainan) Co., Ltd. Batch No. HB8G1021)], while the T-group received tropisetron [(0.1 mg/kg, maximum 2 mg), (Hangzhou Minsheng Pharmaceutical Co., Ltd. Batch No. 20091222)]. Heart rate, blood pressure, pulse oxygen saturation, and heart rhythm were monitored and recorded before and 5 min after drug administration.

Anesthesia induction was performed with propofol (3 mg/kg), fentanyl (2.5 μg/kg), rocuronium (0.6 mg/kg), and dexamethasone (0.15 mg/kg, maximum 5 mg) intravenously. Following tracheal intubation, positive pressure ventilation (PCV) was initiated, and anesthesia was maintained with sevoflurane in 50% oxygen. Before the start of surgery, 1 μg/kg of dexmedetomidine was administered nasally, and 0.5 mg/kg of ketorolac tromethamine (maximum 15 mg) was given intravenously for postoperative pain control. At the end of the procedure, residual neuromuscular blockade was reversed using sugammadex in all patients. The tracheal tube was removed when spontaneous respiration resumed, and the patients were transferred to the post-anesthetic care unit (PACU).

The occurrence and severity of PONV, as well as other adverse reactions, were observed and documented at 6, 24, and 48 h postoperatively. The severity of PONV was graded using a numeric rating scale: “0” for no nausea or vomiting, “1” for nausea without vomiting, “2” for vomiting once within 30 min, and “3” for vomiting two or more times within 30 min (6). If a patient’s PONV score reached “3,” metoclopramide (150 μg/kg) was administered as a rescue medication, and the number of doses and the effectiveness of the rescue medication were recorded. Nausea scoring of children (<6 years) was assessed by a guardian or a follower rather than the child.

**Figure 1:**
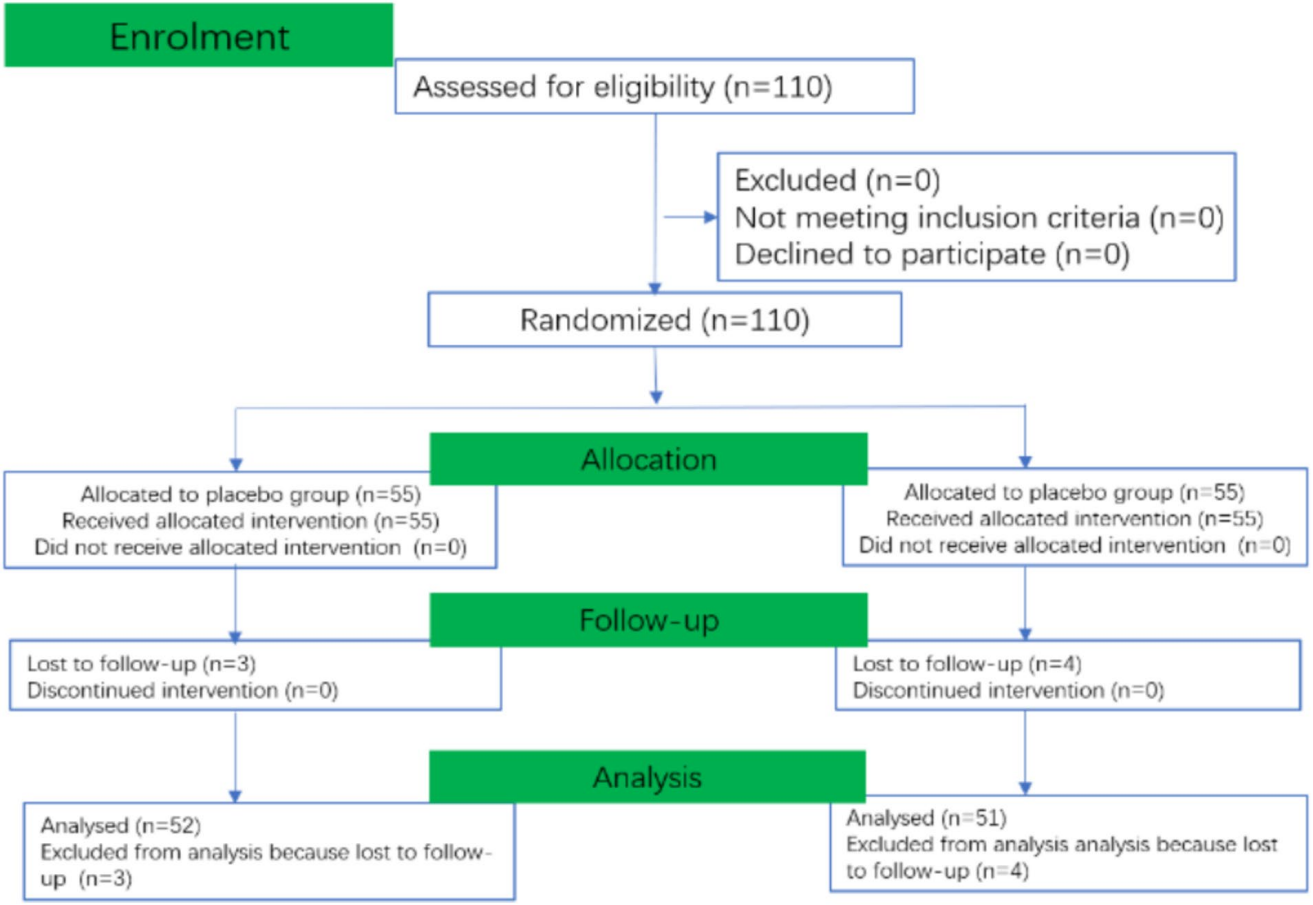
Consort diagram of the study.

**Figure 2:**
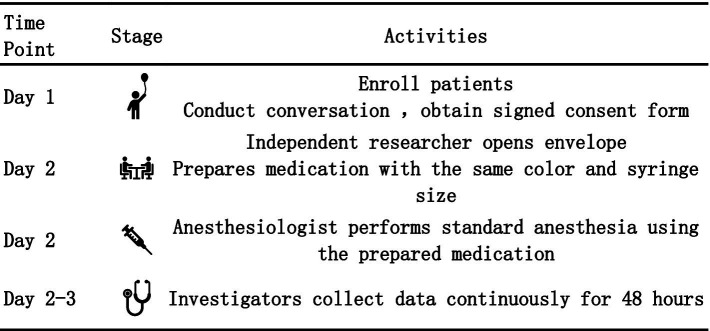
Detailed flow chart.

Data regarding PONV within 24 h after surgery were collected via interviews with nursing staff, patients, and their families. Data from the 24 to 48-h postoperative period were obtained through telephone follow-up interviews. The primary outcome of this study was the incidence of PONV within the 48 h following surgery. Secondary outcomes included the number of children with PONV scores of 1, 2, and 3, the number of children requiring rescue antiemetic therapy, and the assessment of potential adverse events. The consort diagram and detailed flow were shown in Figures 1, 2.

### Sample size determination and statistical methods

Based on a pilot study showing a 28.9% incidence of PONV in children undergoing tonsillectomy and adenoidectomy, we hypothesized that palonosetron plus low-dose dexamethasone would reduce this rate to 10%. The sample size was calculated by PASS (version 15.0.5, NCSS, East Kaysville, UT, USA) with a significance level (two-sided, *α* of 0.05 and statistical power (1-*β*) of 0.8), yielding 52 patients per group. To account for potential loss to follow-up (estimated at 5%), we aimed to recruit 55 patients in each group.

Statistical analyses were performed using SPSS (version 26.0, Chicago, IL, USA) and Microsoft Excel. The Kolmogorov–Smirnov test was used to assess the normality of continuous variables. Data are presented as mean ± SD for continuous variables and as numbers and percentages for categorical variables. Between-group comparisons were conducted using Student’s t-test for continuous variables and either chi-square or Fisher’s exact test, as appropriate, for categorical variables. In the case of non-normally distributed continuous endpoints, the Mann–Whitney U-test was utilized to conduct the comparison. A two-sided *p* value of less than 0.05 was considered statistically significant.

## Results

Of the 110 children enrolled in the study between December 2021 and July 2023, 7 were excluded due to missing data (3 in P-group, 4 in T-group), leaving 103 children for the final analysis. Among these, 52 were assigned to the P-group and 51 to the T-group (Figure 1). Baseline characteristics were similar between the two groups (Table 1).

**Table 1:** Patients’ characteristics.

	P-group (*n* = 52)	T-group (*n* = 51)	*p*
Age (yr)	4.88 ± 1.8	5.50 ± 1.8	0.09
Bodyweight (kg)	21.3 ± 5.8	23.9 ± 9.1	0.09
Height (cm)	113.4 ± 12.3	116.8 ± 13.1	0.11
Gender (M/F)	34/18	30/21	0.10
Duration of surgery (min)	30.5 ± 11.9	29.3 ± 10.7	0.56
Rescue antiemetic (*n*)	0	1	0.31
Dizziness/Headache (*n*)	2	4	0.23
Arrhythmias (*n*)	0	3	0.23

Data are mean ± SD or the number of patients.

In the T-group, one child with a PONV score of 3 within 24 h after surgery required rescue treatment with metoclopramide. The child received an intravenous dose of metoclopramide at 150 μg/kg and was discharged after the symptoms subsided. The difference in the need for rescue antiemetic therapy between the two groups was insignificant (Table 1).

Adverse events, including dizziness, Headache and arrhythmias, were more frequently reported in the T-group than in the P-group, however, the differences were not statistically significant (Table 1).

The incidence of PONV was significantly lower in the P-group compared to the T-group during the 0 to 48-h period (5.8% vs. 25.5%, *p* < 0.05; Table 2). In the T-group, one patient recorded a PONV score of 3 within 24 h after surgery and required rescue therapy. The patient received an intravenous dose of metoclopramide at 150 μg/kg and was discharged after the symptoms subsided.

**Table 2:** Postoperative nausea and vomiting score.

Time	PONV	P-group (*n* = 52)		T-group (*n* = 51)		*p*
		Frequency	%	Frequency	%	
0–6 h	0	0	0	46	90	0.02^#^
	1	0	0	4	7.8	0.03^#^
	2	0	0	1	1.9	0.31
	3	0	0	0	0	
6–24 h	0	50	96	42	78	0.02^#^
	1	0	0	4	7.8	0.03^#^
	2	2	3.8	4	7.8	0.03^#^
	3	0	0	1	1.9	0.31
24–48 h	0	50	96	47	92	0.16
	1	1	1.9	1	1.9	0.98
	2	1	1.9	1	1.9	0.98
	3	0	0	2	3.9	0.14
0–48 h	0	49	94.2	38	74.5	0.005
	1	1	1.9	6	11.8	0.053
	2	3	5.8	7	13.7	0.059
	3	0	0	2	3.9	0.466

^#p < 0.05.^

Although fewer children experienced PONV in the P-group compared to the T-group, there was no statistically significant difference in the distribution of PONV scores of 1, 2, or 3 during the 0 to 48-h postoperative period (Table 2).

The incidence of PONV was significantly lower in the P-group during the 0 to 6-h (0% vs. 10%, *p* < 0.05) and 6 to 24-h postoperative periods (3.8% vs. 7.8%, *p* < 0.05). However, there was no significant difference between the groups during 24-to 48-h (4% vs. 8%, Table 2). The number of children who scored 3 for PONV was not significantly different between the two groups at any postoperative time.

## Discussion

PONV is a common complication in children undergoing adenotonsillectomy. In this randomized trial, we found that the combination of palonosetron and dexamethasone significantly reduced the incidence of PONV to 5.8% during the first 48 h after surgery. This efficacy exceeds that previously reported for ondansetron plus dexamethasone (10.5%) (7) and aligns with findings from Srivastava et al. (8).

The antiemetic effect was particularly pronounced during the first 24 h postoperatively, with no episodes of PONV in the P-group during the first 6 h compared with 10% in the T-group. During the peak period of PONV risk (6–24 h), the incidence was significantly lower with palonosetron than with tropisetron (4% vs. 22%). The low incidence of PONV in both groups between 24 and 48 h (palonosetron, 2% vs. tropisetron, 8%) made it difficult to demonstrate the superiority of either agent during this period.

Previous studies of palonosetron plus dexamethasone reported PONV rates of 9.4 to 43.3% (8–11), considerably higher than our findings. This difference likely reflects our focus on pediatric patients who underwent shorter procedures and received preoperative intranasal dexmedetomidine administration (12, 13). Our results parallel those of Aydin et al. (14), who demonstrated superior efficacy of palonosetron over both ondansetron and tropisetron in adults undergoing middle ear surgery, possibly due to palonosetron’s unique molecular structure and receptor-binding properties (15).

PONV following surgery is an estimated incidence of 20 to 30% in the general surgical population and as high as 80% in high-risk cohorts (3). Given that adenotonsillectomy carries a high risk of PONV and ethical considerations precluded the use of a placebo, we evaluated combination therapy, which inevitably led to some bias in interpreting the experimental results. Our findings support previous evidence that combining palonosetron with dexamethasone provides better PONV prophylaxis than palonosetron alone, which has shown limited efficacy (20% PONV rate) in pediatric strabismus surgery (6). Meta-analyses have demonstrated that combination therapy reduces the need for rescue antiemetics in patients at moderate-to-high risk of PONV (16, 17).

Previous adult studies typically used a fixed dose of 75 μg palonosetron for PONV prevention (11, 18). However, such predetermined dosing, irrespective of body weight, may result in inadequate efficacy or excessive side effects in children. While some studies have evaluated different palonosetron doses in pediatric patients, including a three-arm trial comparing 0.5, 1.0, and 1.5 μg/kg in strabismus surgery (6), the optimal pediatric dosing remains unclear. Our study chose 1 μg/kg based on previous evidence. Although this dose effectively reduced PONV after adenotonsillectomy, complete prevention was not achieved. Further dose–response studies in pediatric adenotonsillectomy are warranted.

PONV in children has multiple risk factors, including age, sex, preoperative anxiety, pain, anesthetic technique, surgical procedure, and perioperative opioid use. To minimize confounding, we restricted enrollment to patients undergoing adenotonsillectomy and standardized the anesthetic protocol, including intraoperative fluid administration. The duration of anesthesia and dosages of anesthetic agents were similar between groups. To reduce opioid-related PONV risk, we used ketorolac for postoperative analgesia, although standardized doses of intravenous fentanyl were administered intraoperatively in both groups.

Both palonosetron and tropisetron provided significant antiemetic effects lasting up to 48 h. Most PONV episodes occurred within the first 48 h after surgery, with palonosetron showing superior efficacy during the 6-to-24-h period, consistent with its known longer duration of action (13). Our follow-up was limited to 48 h postoperatively.

The safety profiles of palonosetron and tropisetron were similar. Common adverse effects of 5-HT_3_-receptor antagonists include Headache, dizziness, arrhythmias, and Q-Tc interval prolongation (19, 20). In our study, the incidences of headache and dizziness were comparable between groups. Although we did not systematically monitor Q-Tc intervals, three patients in the T-group developed a transient junctional rhythm that resolved spontaneously without hemodynamic compromise. While adult studies have shown minimal effects of palonosetron on the Q-Tc interval (21–23), systematic evaluation in pediatric populations is lacking.

Our study has several limitations. First, its single-center design may limit generalizability (24), and the lack of a placebo control group – though ethically necessary – constrains our ability to assess the absolute antiemetic efficacy of palonosetron. Second, we should have systematically monitored Q-Tc intervals. Third, while adequate for the primary endpoint of PONV incidence, our sample size precluded both stratified analyses of outcome differences and comprehensive dose–response evaluation. A definitive dose-finding study would require a larger sample size with escalating doses to establish the plateau of therapeutic effect. Future multicenter studies should address the optimal dosing of palonosetron and evaluate its efficacy in other pediatric surgical procedures.

In conclusion, palonosetron (1 μg/kg) plus dexamethasone provided superior PONV prophylaxis compared with tropisetron plus dexamethasone in children undergoing adenotonsillectomy. This combination appears to be safe and cost-effective in this population. Future studies should evaluate optimal dosing strategies and efficacy in other pediatric surgical procedures.

## Data Availability

The raw data supporting the conclusions of this article will be made available by the authors, without undue reservation.
